# Suicidality in Patients With Epilepsy: Why Should Neurologists Care?

**DOI:** 10.3389/fnint.2022.898547

**Published:** 2022-05-30

**Authors:** Andres M. Kanner

**Affiliations:** Comprehensive Epilepsy Center, Division of Epilepsy, Department of Neurology, Miller School of Medicine, University of Miami, Miami, FL, United States

**Keywords:** major depressive disorder, psychotic disorder, anxiety disorder, suicidal ideation, completed suicide

## Abstract

Suicidality is a relatively common comorbidity in patients with epilepsy (PWE). Population-based studies have revealed lifetime prevalence rates of 25% of suicidal ideation (SI). In addition, PWE without comorbid psychiatric disorders has two to three higher risk of committing suicide and this risk increases by 12- to 32-fold in the presence of various psychiatric disorders. Risk factors are multiple and include socio-demographic, genetic, age and gender, and psychiatric comorbidities. Among the latter, mood, anxiety, and psychotic disorders have been found to be common risk factors for suicidality in PWE, but iatrogenic causes resulting from pharmacotherapy with antiseizure drugs or epilepsy surgery can also cause SI and behavior. Suicidality and epilepsy have a complex bidirectional relation, whereas PWE are at increased risk of suicidality and *vice-versa*. Common pathogenic mechanisms operant in both conditions may explain this bidirectional relation. SI can be easily identified in outpatient epilepsy clinics with screening instruments and can be treated and thus prevent its escalation to suicidal attempts and completed suicide. The aim of this manuscript is to review these data in detail.

## Introduction

Suicidality is a term that encompasses suicidal ideation (SI), behavior, and completed suicide, all of which affect patients with epilepsy (PWE) with a higher frequency than in the general population (Erlangsen et al., 2020). In fact, population-based studies have demonstrated a 2-fold higher risk of completed suicide in PWE in the absence of any psychiatric comorbidity (Christensen et al., 2007). This risk increases in the presence of drug and alcohol abuse mood, anxiety, and personality disorders. Lifetime SI has been identified in one out of every four PWE (Tellez-Zenteno et al., 2007). Unfortunately, SI and behavior go often unrecognized until the patient had a complete or attempted suicide.

There is a complex relationship between suicidality and epilepsy, part of which is manifested by their bidirectional relation and shared pathogenic mechanisms. The purpose of this article is to review the epidemiologic, clinical manifestations, pathogenic mechanisms, and strategies that clinicians can consider to identify patients at risk of suicide.

### Clinical Expressions of Suicidality

Suicidal ideation and behavior can be the expression of interictal, peri-ictal, and iatrogenic phenomena. Interictal SI and behavior are temporally independent of seizures, while peri-ictal SI and behavior preceed (pre-ictal), are the clinical expression of the ictal activity or follow the occurrence of seizures (postictal). While interictal psychiatric symptoms are investigated and recognized by clinicians, peri-ictal symptoms are not, despite their relatively high prevalence in patients with treatment-resistant epilepsy (Kanner et al., 2004; Subota et al., 2019). For example, in a study of 100 consecutive patients with treatment-resistant focal epilepsy, habitual postictal symptoms of depression and anxiety were identified in 43 and 45%, respectively, while 13% endorsed postictal SI after more than 50% of seizures, with a median duration of 24 h (range 6–108 h). Eight patients experienced passive and five active SI (Kanner et al., 2004).

Iatrogenic suicidality can result from the introduction or discontinuation of certain antiseizure drugs (ASDs) with negative and positive psychotropic properties, respectively, in patients with a personal and/or family history of psychiatric disorders (Brent et al., 1987; Mula et al., 2009; Kanner, 2016; Josephson et al., 2019). In addition, suicidality can also be an iatrogenic complication of epilepsy surgery (Salanova et al., 2002; Hamid et al., 2011). Of note, the same patient may exhibit interictal, peri-ictal, and iatrogenic suicidality (Kanner et al., 2004).

The clinical manifestations of suicidality have been standardized in The Columbia Classification Algorithm of Suicide Assessment (Posner et al., 2007). These include:

*Suicidal ideation* refers to thoughts of suicide or longing to be dead. These can be divided into *passive and active* SI. Passive SI includes thoughts of wishing to be dead, but no plans or actions have been entertained. *Active SI* implies the intention of committing suicide and the consideration of a plan but has not performed any preparatory activities.

*Suicide attempts* refer to non-fatal, potentially self-harmful behavior with an intent to die, which can be inferred from the patient’s act, even if they do not lead to physical harm.

*Preparatory actions* refer to the planning, but not the implementation of the suicide plan.

C*ompleted suicide* is defined as a fatal self-injurious act with evidence of at least some intent to die. Suicide can result from an acute and reactionary act rather than a carefully planned act.

Of note, not all self-injurious acts are the expression of a suicidal attempt. Often, patients experience a relief of tension and/or distress (e.g., cutting in patients with bipolar disorders). In addition, patients may recur to these acts to attract the attention of others.

### Epidemiology

Suicide has been attributed as the cause of death in approximately 1.5% of total deaths in the world, according to the Global Burden of Disease Study 2016, which included statistics from 195 countries (Naghavi, 2019). It was one of the 10 most common causes of death in 5 of 21 defined regions, which included low- and middle-income nations where the risk of epilepsy is higher (Beghi et al., 2019).

As stated above, SI and behavior are more common in PWE than in the general population. For example, in a population-based study from Canada, lifetime SI was endorsed by 25% of PWE compared to 13.3% of controls (Tellez-Zenteno et al., 2007). Likewise, completed suicide was 3-fold higher in PWE than in controls (2.32 *vs*. 0.74%) in another population-based study (Christensen et al., 2007). Of note, the absence of comorbid psychiatric disorders does not preclude the higher suicidal risk (rate ratio [*RR*]: 3.17; 95% *CI*: 2.88–3.50), which significantly increases their RR in their presence to 13.7 (95% *CI*: 11.8–16.0) (Christensen et al., 2007). A review of 74 studies that investigated the death by suicide in PWE found a standardized mortality ratio (SMR) of 3.3 (95% *CI*: 2.8–3.7) (Bell et al., 2009). The SMR was significantly increased in people with an incident or newly diagnosed epilepsy (SMR 2.1), as well as in those with prevalent epilepsy (SMR 4.8).

#### Bidirectional Relation Between Suicidality and Epilepsy

Understanding the epidemiologic aspects of suicidality in PWE cannot be complete without factoring in the bidirectional relation between suicidality and epilepsy, where by not only PWE are at increased risk of developing SI and behavior, but patients with suicidality also are at increased risk of developing epilepsy. In a population-based study, Hesdorffer et al. (2012) found that the incidence risk ratio of suicide was elevated for 3 years prior (3.1–4.5) and the first year following the diagnosis of epilepsy. Bidirectional relations were also identified between epilepsy and other psychiatric disorders that are associated with an increased suicidal risk such as mood, anxiety, and psychotic disorders (Hesdorffer et al., 2012). In one population-based study, the hazard of epilepsy following incident depression was 2.55 times (95% *CI*: 2.49–2.66) higher than that of the general population and was 9.85-fold higher (95% *CI*: 5.74–16.90) among patients with a more severe form of depression (Josephson et al., 2017). In another population-based study, the incidence rate for psychosis, anxiety, and substance abuse were higher than those of controls over the 3 years prior to and the subsequent 2–3 years following an incident diagnosis of epilepsy (Hesdorffer et al., 2012).

While we cannot exclude the possibility of unwitnessed and/or unrecognized (non-motor) seizures occurring prior to the diagnosis of epilepsy, the existence of common neurobiologic pathogenic mechanisms operant in these psychiatric disorders and epilepsy have been postulated as a possible explanation of these bidirectional relations and are reviewed in a section below.

### Risk Factors

#### Psychosocial Factors

These factors have been identified in the general population as well as in PWE. These include traumatic experiences in childhood such as physical and sexual abuse, parental substance abuse and criminality, socio-economic deprivation, all of which have been associated with a cumulative effect on suicide risk (Björkenstam et al., 2017). In addition, people at risk of stigma, such as lesbian, gay, bisexual, transgender, and indigenous people have been also found to be at increased risk of suicidal behavior (King et al., 2009; Haas et al., 2011; Björkenstam et al., 2017). The economic status of PWE can also play a role in the suicidality risk as demonstrated in a Taiwanese study in which patients with low and medium incomes had an increased risk of suicide attempts (adjusted hazard ratio [aHR]: 1.75 [95% *CI*: 1.25–2.45] and 2.07 [95% *CI*: 1.52–2.82], respectively) (Harnod et al., 2018).

#### Age and Gender

The risk of completed suicide, increases with age in PWE as well as in the general population. For example, completed suicide was identified four-times more among PWE aged 30–64 than those aged between 10 and 29 (Tian et al., 2019). While patients in their middle ages have the highest risk, it decreases among patients over 65 years (Harnod et al., 2018). Likewise, the risk of completed suicide is significantly higher for those who developed epilepsy between the ages of 0–9 (*RR*: 12; 95% *CI*: 2.6–55.7) and 10–17 (*RR*: 19.6; 95% *CI*: 4.8–80.5) compared to age ≥ 30 years (Nilsson et al., 2002).

With respect to gender, the risk of completed suicide is twice as higher in men than women, but this difference is less apparent in urban areas (Nilsson et al., 2002).

#### Genetic Predisposition

Suicide behavior is more frequent in monozygotic than dizygotic twins (Tidemalm et al., 2011), while having a family history of suicide increases the risk of suicidal behavior, but not ideation by 3–10 fold (Turecki, 2014).

#### Psychiatric History

Suicide attempts are the strongest predictors for completed suicide in patients with and without epilepsy (World Health Organisation, 2020). Not surprisingly, psychiatric history is common in people with SI and behavior. For example, 90% of 3,275 patients without epilepsy who committed suicide had been diagnosed with a psychiatric disorder before their death (Arsenault-Lapierre et al., 2004). Major depression and bipolar disorder were the most frequent psychiatric disorders and accounted for 50% of death by suicide (Holma et al., 2014), while isolated anxiety disorders and mixed depression and anxiety were found to raise the odds of suicide by 2- and 17-fold, respectively (Sareen et al., 2005; Bolton et al., 2008), and lifetime psychotic episodes raise the hazard of completed suicide by over 9-fold (Sharifi et al., 2015). Yet, in people without epilepsy, personality disorders associated with impulse control disorders, antisocial behavior, and substance abuse have been found to be even stronger predictors of a first suicide attempt in adolescents and young adults (Séguin et al., 2014), while borderline personality disorder is associated with a 4-fold higher risk of suicidal behavior than other psychiatric disorders (Séguin et al., 2014).

As in patients without epilepsy, mood and anxiety disorders have been found to be strong predictors of SI in PWE (Jones et al., 2003; Mula et al., 2010; Hecimovic et al., 2012; Altura et al., 2016). Furthermore, in a population-based study from Denmark, mood disorders increased the risk of completed suicide by 32 fold (20.8–49.4), anxiety disorders by 11.4 fold, schizophrenia by 12.5 fold, chronic alcohol use by 20.1 fold and other psychiatric disorders by 22.0 fold (Christensen et al., 2007).

Postictal psychotic episodes correspond to 25% of all psychotic disorders of epilepsy and affect 2% of PWE (Clancy et al., 2014) and are associated with suicide attempts in up to 7% of episodes (Kanemoto et al., 2010). Psychotic symptoms typically occur following an asymptomatic period of 12–24 h after the last seizure (Logsdail and Toone, 1988; Kanner et al., 2004).

In contrast to people without epilepsy, the impact of personality disorders on suicidality has yet to be investigated in population-based studies in PWE.

#### Epilepsy and Seizure-Related Factors

For a long time, an increased suicidality risk had been associated with temporal lobe epilepsy (TLE) as suggested by a meta-analysis of studies that investigated the risks of completed suicide in PWE and which yielded a SMR of 6.57 (95% *CI*: 1.79–16.8) (Bell et al., 2009). On the other hand, a case-control study from Denmark found a relative risk of 0.6 (95% *CI*: 0.2–1.7) for temporal *versus* generalized epilepsy (Nilsson et al., 2002). Thus, the types of epilepsy and seizure frequency have not been associated with a higher risk of completed suicide (Nilsson et al., 2002; Park et al., 2015).

On the other hand, completed suicide has been identified with an increasing frequency 6 months after the diagnosis of epilepsy in patients with and without psychiatric comorbidity. Thus, in the population-based study from Denmark cited above, adults with a psychiatric history had a 29-fold higher risk of completed suicide, while in those without any psychiatric disorder the risk was 5-fold higher (Christensen et al., 2007). This risk can be also noted in the reported increased risk of SI requiring hospitalization within 90 days of an admission for epilepsy (RR: 4.91 [95% *CI*: 3.83–6.27]) compared to that for a stroke or a combination of chronic obstructive pulmonary disease, urinary tract infections, and pneumonia (RR: 2.66 [95% *CI*: 2.23–3.05]) (Xu et al., 2018).

The lifetime history of SI does not appear to be higher among patients with treatment-resistant epilepsy than that of a general population of PWE. For example, the lifetime prevalence of SI was 25% in the Canadian population study cited above (Tellez-Zenteno et al., 2007) and 12.1–14.1% among patients with treatment-resistant focal epilepsy (Hesdorffer et al., 2013). On the other hand, the lifetime prevalence of suicidal attempts was higher among patients with treatment-resistant focal epilepsy (13.1%) than those with newly diagnosed focal epilepsy (5%).

#### Suicidality as an Iatrogenic Effect

*Antiseizure medications and suicide:* In 2008, the United States Food and Drug Administration requested from all ASD manufacturers for the insertion of a black box warning about SI and completed suicide (FDA, 2008). This decision was based on the incidence of SI and suicidal behavior during multicenter, double-blind, placebo-controlled trials of 11 ASDs that were submitted to the FDA for approval and which found SI or behavior in 0.37% of patients randomized to the ASD *vs*. 0.24% in those given placebo. Yet, subsequent reviews of the methodology used by the FDA and its analysis of the data revealed multiple flaws (Hesdorffer and Kanner, 2009). First, the FDA relied only on the spontaneous report of these symptoms, rather than asking for an inquiry of these symptoms in all patients. Accordingly, patients with SI and behavior that failed to report spontaneously these symptoms went undetected. In fact, the suicidality prevalence rates reported by the FDA is significantly lower than the 1.6–3.9% recent high-risk SI and 1.0–4.7% recent suicide attempt in a study of patients with treatment-resistant epilepsy that would be considered for ASD trials (Hesdorffer et al., 2013). Secondly, the FDA grouped all ASDs as a single class of drugs with respect to the suicidality risk even though their own data revealed that several ASDs (carbamazepine and valproic acid) had a protective effect. Thirdly, the FDA did not factor in the role of a prior psychiatric (and suicidality) history in their findings. Not surprisingly, subsequent studies reported contradictory findings to those of the FDA and among the studies (Simon et al., 2006; Olesen et al., 2010; Patorno et al., 2010; Pugh et al., 2013). Furthermore, the bidirectional relation between suicidality and epilepsy must be taken into consideration when interpreting any impact of an ASD on the suicidality risk (Hesdorffer et al., 2012).

Yet, there is significant evidence that several ASDs have negative psychotropic properties which can lead to the development of psychiatric symptoms, including SI. These include, barbiturates (phenobarbital and primidone), levetiracetam, topiramate, zonisamide, vigabatrin, tiagabine, and perampanel (Brent et al., 1987; Mula et al., 2009; Kanner, 2016; Josephson et al., 2019; Kanner et al., 2021). Yet, these adverse events are more likely to occur in patients with a previous psychiatric history and/or a family psychiatric history (Brent et al., 1987; Mula et al., 2009; Josephson et al., 2019; Kanner et al., 2021).

Conversely, several ASDs have mood stabilizing (carbamazepine, oxcarbazepine, valproic acid, and lamotrigine), antidepressant (lamotrigine), and anxiolytic (gabapentin, pregabalin, and benzodiazepines) properties, and their discontinuation can result in the recurrence of mood and/or anxiety disorders in patients with a prior psychiatric history, which had remitted while they were taking these ASDs (Kanner, 2006).

### Suicidality as an Iatrogenic Effect of Epilepsy Surgery

Temporal and extratemporal resections for treatment-resistant focal epilepsy have been reported to cause *de novo* mood, anxiety, and psychotic disorders as well as recurrence and exacerbation of preoperative psychiatric disorders in 18–46% of patients (Wilson et al., 2001; Nilsson et al., 2003; Wrench et al., 2004; Hellwig et al., 2012). Mood and anxiety disorders are the most frequently seen in approximately 30% of patients during the first 6 months after surgery, which remit in most, but not all patients after 12 months. Likewise, high SMRs following temporal (SMR 13.9) and extratemporal (SMR 6.4) resections were found in a meta-analysis of 76 studies (Bell et al., 2009). On the other hand, whether the post-surgical seizure outcome has any impact on the suicidality risk is yet to be established as several studies reported variable findings (Wilson et al., 2001; Nilsson et al., 2003; Wrench et al., 2004).

The concept of ‘the burden of normality’ has been proposed as a potential explanation for post-surgical psychopathology, such as SI and behavior (Wilson et al., 2001). This theory postulates that patients are unable to face the responsibilities of life after reaching seizure-freedom as they were not prepared educationally or vocationally because of the intractable seizure disorder. These patients relied on the social assistance for their economic support, which is likely to be withdrawn upon the achievement of seizure-freedom. In fact, several studies have revealed that working before surgery is the strongest predictor of working after surgery. These phenomena can be complicated by post-surgical cognitive complications (worsening of verbal memory after resections in the dominant hemisphere) or post-surgical depression and/or anxiety disorders (Hellwig et al., 2012).

## Common Pathogenic Mechanisms Between Suicidality and Epilepsy

Neurobiologic pathogenic mechanisms operant in epilepsy and suicidality may explain the bidirectional relation between the two conditions. These include: (i) disturbances of common neurotransmitters, such as decreased serotonergic and GABAergic activity and increased glutamatergic activity. (ii) Hyperactive hypothalamic–pituitary–adrenal axis (HPAA) resulting in high serum cortisol concentrations. (iii) Decreased neurotrophins (BDNF) (iiii) Neuro-immune dysfunction, including increased cytokine secretion. For detail reviews by Kanner (2006) and Hecimovic et al. (2021).

A decreased serotonergic activity is the best well-known neurotransmitter disturbance associated with suicide since 1972 and has been demonstrated in preclinical and clinical studies in PWE (Kanner, 2006; Hamid and Kanner, 2013; Kanner et al., 2018). Indeed, low concentrations of the serotonin (5HT) metabolite, 5-hydroxyindoleacetic acid (5-HIAA), have been identified in the cerebrospinal fluid of individuals who died of violent suicides (Mann, 2003). These findings have been replicated in suicide and more lethal but non-fatal suicide attempts, together with low levels of 5-HT and/or 5-HIAA in the brainstem serotonin nuclei and altered serotonin receptor and serotonin transporter binding in brains of patients who died of suicide (Lester, 1995).

Glutamate and GABA are two neurotransmitters with pivotal pathogenic roles in epilepsy. High glutamatergic and low GABAergic activity mediate the cortical hyperexcitability and epileptogenesis. Likewise, high glutamate and low GABA concentrations have been identified in magnetic resonance spectroscopy studies (1H-MRS) in patients with primary mood disorders (Sanacora et al., 1999; Hashimoto et al., 2007).

The role of a hyperactive hypothalamic–pituitary–adrenal axis (HPAA) in suicidality has well been established, as evidenced by high cortisol serum concentrations in patients who died from suicide (Kanner, 2006; Hecimovic et al., 2021). Furthermore, a hyperactive HPAA has been identified in the comorbid psychiatric disorders associated with suicidality such as mood, anxiety disorders, and chronic stress (Sapolsky, 2000; Kanner, 2006; Hecimovic et al., 2021). For example, the non-suppression of cortisol in response to the dexamethasone administration test (DST), is a measure of the HPAA function. The DST cortisol non-suppressors were found to have a 4.5-fold risk of dying by suicide. Of note, high cortisol serum concentrations have correlated with low CSF 5HIAA in patients who had attempted suicide.

The role of a hyperactive HPAA has been also recognized in TLE. High cortisol concentrations can cause damage to hippocampal neurons, particularly CA3 pyramidal neurons, through a reduction of dendritic branching and a loss of dendritic spines that are included in glutamatergic synaptic inputs (Sapolsky, 2000; Kumar et al., 2007).

Finally, neuroimmune dysfunction has also been postulated as a common pathogenic mechanism operant in suicidality and its comorbid psychiatric disorders and in epilepsy (Vezzani et al., 2008; Black and Miller, 2015; Brundin et al., 2017; Kanner et al., 2018; Hecimovic et al., 2021). The release of proinflammatory cytokines such as interferon-α (IFN-α), tumor necrosis factor-α (TNF-α), IL-6, and IL-1β are pivotal players in epilepsy and psychiatric disorders (Vezzani et al., 2008; Hecimovic et al., 2021).

## Screening for Suicidality in the Epilepsy Clinic

Given the relatively high prevalence of SI and behavior and the increased risk of complete suicide in PWE, it is essential that screening for suicidality be an integral part of the evaluation of any of these patients, at the time of the first visit and on subsequent visits. Such screening must not be limited to suicidality symptoms but should include the common psychiatric comorbidities that are associated with them, particularly mood and anxiety disorders.

Self-rating screening instruments can facilitate the screening of suicidality and the common psychiatric comorbidities (e.g., mood and anxiety disorders) associated with it in outpatient epilepsy/neurology clinic. The Neurological Disorders Depression Inventory for Epilepsy (NDDIE) (Gilliam et al., 2006) is the preferred instrument to screen for major depressive episode; this instrument was specifically developed for PWE. The Generalized Anxiety Disorder-7 (GAD-7) can be used for generalized anxiety and panic disorders (Spitzer et al., 2006). To screen for suicidality, clinicians can use item #4 of the NDDIE (‘I would be better off dead’). When compared to the suicidality module of the Mini International Neuropsychiatric Inventory (MINI), it showed excellent discrimination and specificity (91%; 95% *CI*: 87.4–93.6), with reliable sensitivity (84%; 95% *CI*: 60.4–96.6) and positive (9.21; 95% *CI*: 6.3–13.5) and negative likelihood ratios (0.17; 95% *CI*: 0.06–0.50) (Sheehan et al., 1998). An alternative is the item #9 of the Beck Depression Inventory-II (Beck, 1996), which breaks down SI into passive and active and hence provides more detail. Patients who score in the symptomatic range should then be referred for a formal evaluation with a mental health practitioner.

For research purposes, an evaluation with a structured clinical interview is preferred. The Structured Clinical Interview for DSM-IV (SCID) is considered the gold standard for identifying comorbid discrete psychiatric diagnoses, SI, and suicidal behavior. This interview must be administered by a trained research assistant. An alternative is the MINI, which also identifies Axis I diagnoses and has a suicidality module (Sheehan et al., 1998), which can be used in the outpatient epilepsy clinics. The Columbia Suicide Severity Rating Scale (CSSRS) has become the screening instrument required for regulatory trials by the FDA (Posner et al., 2007). It also requires training to ensure inter- and intra-rater reliability.

## Suicide Prevention

A timely identification of patients at risk of suicidal behavior is the best way to prevent suicide. Accordingly, it falls upon the neurologist or internist treating the PWE to screen for suicidality symptoms and the common psychiatric conditions associated with it (e.g., mood and anxiety disorder) given that 30% of those expressing SI will eventually make and implement a plan (Nock et al., 2013). The use of screening instruments is, therefore, a first step to identify patients at risk for suicidality in an outpatient epilepsy/neurology clinic.

The utility of screening instruments by epileptologists is illustrated in a study conducted in the outpatient epilepsy clinic of the Rush Epilepsy Center in Chicago, IL, United States. Its aim was to establish whether the use of screening instruments for MDE, GAD, and suicidality can translate into their improvement in a busy outpatient epilepsy clinic (Kanner et al., 2014, data presented at the annual meeting of the American Epilepsy Society). To that end, every adult, age 18 years and older seen at the Epilepsy Clinic were asked to complete the NDDI-E, the GAD-7, and the suicidality module of the MINI at every visit. The treating epileptologists had to decide on an intervention for patients with NDDI-E scores > 15 (suggestive of a probable MDE), GAD-7 > 10 (suggestive of a probable generalized anxiety disorder) and any positive suicidality symptom on the suicidality module of the MINI. Interventions included referral to a mental health professional, start or modify a pharmacologic regimen with psychotropic drugs, or other interventions. A total of 635 PWE completed these screening instruments. Among these patients, 115 had been seen in two consecutive visits and were included in this study that investigated (i) the number of PWE who met the criteria for possible MDE, possible GAD, possible MDE + GAD and any suicidality symptom on visit 1 who became asymptomatic on visit 2 and (ii) the number of patients who were not symptomatic on visit 1 and became symptomatic on visit 2. Visit 2 occurred after a mean period of 122 days (± 77 days). The results are summarized in Table 1.

**TABLE 1:** Changes of psychiatric symptoms from visit 1 to visit 2.

Psychiatric Dx *N* = 155	Symptomatic on visit 1 (%)	Symptomatic on visit 2 (%)	Improved (%)	*De novo* symptomatic on visit 2 (%)
Only MDE	10 (6.5)	4 (2.6)	6 (60)	3 (2.6)
Only GAD	13 (8.4)	6 (3.9)	7 (47)	5 (4.3)
MDE + GAD	17 (11)	5 (3.2)	12 (66)	2 (1.7)
Suicidal ideation*	21 (13.5)	6 (3.9)	15 (71.5)	9 (7.8)
Suicidal attempt*	2 (1.3)	0	2 (100)	0
Total	40 (26)	15 (9.7)	25 (62.5)	10 (6.5)

*Patients with SI and attempt were also experiencing MDE and/or GAD.

These data revealed the presence of MDE and/or GAD with or without suicidality symptoms in one out of every four outpatients in visit 1 and clearly demonstrate that epileptologists can successfully identify symptomatic patients and make an intervention that resulted in a symptom remission of two-thirds of symptomatic patients. These data also demonstrate the need to administer the screening instruments at every visit as close to 7% patients may become symptomatic *de novo* in the follow-up visit.

In PWE with SI, neurologists can estimate the urgency for a psychiatric intervention by asking the patient “What has kept you from acting on your thoughts?” Patients with suicidal behavior should be evaluated by a mental health professional without delay, particularly those with recent suicide attempts or previous suicide attempts who are suffering from a comorbid mood and/or anxiety disorder and endorse SI (Practice guideline for the assessment and treatment of patients with suicidal behaviors, Am J Psychiatry, 2003; Zalsman et al., 2016). In addition to the recognition of comorbid psychiatric disorders, it is essential to investigate any potential precipitating factors including any new medical comorbidities, loss of gainful employment or poor performance in school, loss of a family member, partner, or a key person in their support network, social isolation, sexual and gender identity crises, and exposure to or persistence of an abusive relationship. Furthermore, the involvement of family members and other sources of support are of the essence.

In PWE, an iatrogenic cause of the SI/behavior must be ruled-out either if it followed the introduction of an ASD with negative psychotropic properties (e.g., levetiracetam, topiramate, zonisamide, brivaracetam, and perampanel) or the discontinuation of an ASD with positive psychotropic properties (e.g., carbamazepine, oxcarbazepine, valproic acid, and lamotrigine) that was keeping the patient’s psychiatric disorder in remission (Kanner, 2016). In the former case, a rapid switch-over to another ASD, or the discontinuation of the offending drug should be conducted in an inpatient setting (if possible, with EEG monitoring) to minimize the risk of seizures resulting from its discontinuation. In the latter, the ASD that was discontinued should be restarted or replaced by another with similar psychotropic properties.

Most of the psychotropic drugs used in patients without epilepsy can be considered for PWE, as most antidepressants and antipsychotics are safe in these patients when used at therapeutic doses (Kanner, 2016). Pharmacotherapy should be combined with psychotherapy, including cognitive behavior therapy and/or group therapy and/or family therapy. While clozapine has shown efficacy in alleviating suicidal behavior, it should be used as a last resort in PWE because of its proconvulsant properties and its serious adverse effects (Devinsky et al., 1991). Finally, electroconvulsive therapy is effective in the treatment of severe suicidality in people without epilepsy and can be used as well in PWE (Lunde et al., 2006).

## Concluding Remarks

Suicidality is a very serious comorbidity in PWE, which occurs with a higher frequency than in the general population. Suicidality and epilepsy have a bidirectional relation that results from common pathogenic mechanisms operant in both conditions. Routine screening for suicidal behavior should be conducted in every PWE and in patients found to display suicidality symptoms their cause should be thoroughly investigated, including iatrogenic causes. Non-pharmacologic and pharmacologic interventions should be considered, and psychotropic drugs should not be withheld since, with few exceptions, they are safe in PWE when prescribed at recommended doses. Collaborations between neurologists and psychiatrists are needed to further understand the epidemiology and risk factors of suicidal behavior in epilepsy, which will inevitably lead to robust and valid prediction models and evidence-based prevention strategies.

## Author Contributions

The author confirms being the sole contributor of this work and has approved it for publication.

## Conflict of Interest

The author declares that the research was conducted in the absence of any commercial or financial relationships that could be construed as a potential conflict of interest.

## Publisher’s Note

All claims expressed in this article are solely those of the authors and do not necessarily represent those of their affiliated organizations, or those of the publisher, the editors and the reviewers. Any product that may be evaluated in this article, or claim that may be made by its manufacturer, is not guaranteed or endorsed by the publisher.
